# Group I mGluR-dependent depotentiation in the lateral amygdala does not require the removal of calcium-permeable AMPA receptors

**DOI:** 10.3389/fnbeh.2014.00269

**Published:** 2014-08-08

**Authors:** Kyungjoon Park, Sukwoon Song, Ingie Hong, Beomjong Song, Jeongyeon Kim, Sungmo Park, Junuk Lee, Sangho Song, Bobae An, Jihye Kim, C. Justin Lee, Ki Soon Shin, Sukwoo Choi, Sukwon Lee

**Affiliations:** ^1^School of Biological Sciences, College of Natural Sciences, Seoul National UniversitySeoul, Korea (ROK); ^2^The Solomon H. Snyder Department of Neuroscience, Howard Hughes Medical Institute, Johns Hopkins University School of MedicineBaltimore, MD, USA; ^3^Institute of Neuroscience, Technical University of MunichMunich, Germany; ^4^Center for Neural Science and Center for Connectomics, Korea Institute of Science and TechnologySeoul, Korea (ROK); ^5^Department of Biology, Department of Life and Nanopharmaceutical Sciences, Kyung Hee UniversitySeoul, Korea (ROK)

**Keywords:** calcium-permeable AMPA receptors, synaptic depotentiation, fear conditioning, lateral amygdala, long-term depression

## Abstract

There is conflicting evidence regarding whether calcium-permeable receptors are removed during group I mGluR-mediated synaptic depression. In support of this hypothesis, AMPAR rectification, a correlative index of the synaptic expression of GluA2-lacking calcium–permeable AMPARs (CP-AMPARs), is known to decrease after the induction of several types of group I mGluR-mediated long-term depression (LTD), suggesting that a significant proportion of synaptic CP-AMPARs is removed during synaptic depression. We have previously demonstrated that fear conditioning-induced synaptic potentiation in the lateral amygdala is reversed by group 1 mGluR-mediated depotentiation. Here, we examined whether CP-AMPARs are removed by mGluR1-mediated depotentiation of fear conditioning-induced synaptic potentiation. The synaptic expression of CP-AMPARs was negligible before, increased significantly 12 h after, and returned to baseline 48 h after fear conditioning, as evidenced by the changes in the sensitivity of lateral amygdala synaptic responses to NASPM. Importantly, the sensitivity to NASPM was not altered after induction of depotentiation. Our findings, together with previous results, suggest that the removal of CP-AMPARs is not required for the depotentiation of fear conditioning-induced synaptic potentiation at lateral amygdala synapses.

## Introduction

Calcium-permeable AMPA receptors (CP-AMPARs) are expressed transiently in excitatory neuron synapses but have been shown to be rapidly removed upon group 1 metabotropic glutamate receptor (mGluR) stimulation (Bellone and Lüscher, 2005, 2006; Clem and Huganir, 2010; McCutcheon et al., 2011; Loweth et al., 2013). Group 1 mGluR-induced synaptic depression also reduces the CP-AMPAR-mediated current in GABAergic neurons (Kelly et al., 2009), suggesting that group 1 mGluRs may specifically target these CP-AMPARs for synaptic removal. However, mGluR-LTD has been described in a wide variety of synaptic circuits that generally lack pre-existing CP-AMPAR expression (for review, see Lüscher and Huber, 2010). Moreover, some types of mGluR-mediated synaptic depression require the phosphorylation/dephosphorylation of the AMPAR subunit GluA2 (Chung et al., 2003; Moult et al., 2006), which CP-AMPARs generally do not contain. Thus it is unclear whether group 1 mGluR-mediated synaptic depression requires, or preferentially recruits, CP-AMPAR removal.

The lateral amygdala (LA) is known to be critical for fear memory acquisition, extinction and renewal (LeDoux, 2000; Maren and Quirk, 2004; Kim et al., 2007; Myers and Davis, 2007; Knapska and Maren, 2009; Lee et al., 2013). The thalamic input synapses onto the LA (T-LA synapses) have been extensively studied as a site of learning-induced plasticity, and T-LA synaptic efficacy is tightly correlated with fear memory strength (McKernan and Shinnick-Gallagher, 1997; Rogan et al., 1997) Among several forms of synaptic plasticity at T-LA synapses, long-term depression (LTD) and/or depotentiation have been proposed as a cellular mechanism underlying extinction (or reconsolidation update) of fear memory (Lin et al., 2003, 2005; Kim et al., 2007; Clem and Huganir, 2010). LTD is a *de novo* decrease in synaptic efficacy, whereas depotentiation represents a net return of the potentiated synaptic efficacy to baseline (Collingridge et al., 2010). Although both alterations result in a decrease in synaptic efficacy, the underlying mechanisms of these two types of plasticity may be different (Wagner and Alger, 1996; Kulla et al., 1999; Klausnitzer et al., 2004).

Several previous studies have reported that fear conditioning-induced synaptic potentiation *in vivo* at T-LA synapses can be depotentiated in brain slices prepared from conditioned animals (Kim et al., 2007; Clem and Huganir, 2010, 2013). Our previous study has shown that depotentiation is blocked by intracellular dialysis of the GluA2_3Y_ peptide, which prevents the internalization of GluA2-containing AMPARs (Ahmadian et al., 2004). Moreover, fear extinction reverses the conditioning-induced enhancements in the surface expression of synaptic GluA2 at LA synapses and occludes depotentiation, suggesting mutual mechanisms. Together, these findings suggest that depotentiation at T-LA synapses involves the internalization of GluA2-containing and, thus, calcium-impermeable AMPARs. However, conflicting evidence has also been presented primarily based on AMPAR rectification, an index of the synaptic expression of CP-AMPARs, as GluA2-lacking CP-AMPARs are removed during a type of LTD whose magnitude increases after fear conditioning (i.e., depotentiation-like plasticity) at T-LA synapses (Clem and Huganir, 2013). One compromising factor in the latter study is that depotentiation could not be studied in isolation because the same stimuli also induces LTD before fear conditioning. Therefore, the specific subtypes of AMPARs involved in depotentiation of fear conditioning-induced synaptic potentiation are somewhat unclear.

In this study, we used the sensitivity of T-LA synaptic responses to 1-naphthylacetyl spermine (NASPM), a CP-AMPAR antagonist, as an index of the synaptic expression of CP-AMPARs to determine whether CP-AMPARs are removed during depotentiation of fear conditioning-induced potentiation under conditions in which the depotentiation of fear conditioning-induced synaptic potentiation can be examined in isolation.

## Materials and methods

### Animals and auditory fear conditioning

All procedures were approved by the Institute of Laboratory Animal Resources of Seoul National University (Korea). Male Sprague-Dawley rats (4–5 weeks old) were maintained with free access to food and water under an inverted 12/12 h light/dark cycle (lights off at 09:00 h). Behavioral training was done during the dark portion of the light/dark cycle. For fear conditioning, rats were placed in a conditioning chamber and left undisturbed for 2 min. Then, a neutral tone (30 s, 2.8 kHz, 85 dB) coterminating with an electrical foot shock (1.0 mA, 1 s) was presented three times at an average interval of 100 s. After fear conditioning, rats were returned to their home cages until preparation of brain slices. Rats in naïve groups stayed in their home cages until brain slices were prepared.

### Brain slice preparation

Sprague-Dawley rats (4–5 weeks old) were anesthetized with isoflurane and decapitated. Whole brains were isolated and placed in an ice-cold modified aCSF solution containing (in mM) 175 sucrose, 20 NaCl, 3.5 KCl, 1.25 NaH2PO4, 26 NaHCO3, 1.3 MgCl2, 11 D-(+)-glucose, and was gassed with 95% O_2_/5% CO_2_. Coronal slices (300 μm) including the LA were cut using a vibrating blade microtome (VT1200S, Leica Biosystems, Germany) and incubated in normal aCSF containing (in mM) 120 NaCl, 3.5 KCl, 1.25 NaH2PO4, 26 NaHCO3, 1.3 MgCl2, 2 CaCl2, 11 D-(+)-glucose, and was continuously bubbled at room temperature with 95% O_2_/5% CO_2_. Just before a given slice was transferred to the recording chamber, the cortex overlying the LA was cut away with a scalpel, so the addition of picrotoxin (100 μM; Abcam Plc., UK) would block cortical epileptic burst discharges from invading the LA.

### Afferent stimulation and recording conditions

We chose brain slices containing a well-isolated, sharply defined trunk (containing thalamic afferents) crossing the dorsolateral division of the LA where the somatosensory and auditory inputs converge. The sizes of the LA and the central amygdala were relatively constant in the utilized slices; when multiple trunks were observed, we used the closest trunk to the central nucleus of the amygdala. Unless otherwise noted, the thalamic afferents were stimulated at a frequency of 0.067 Hz using a concentric bipolar electrode (CBAEC75; FHC Inc., USA). The stimulation electrode was placed at the midpoint of the trunk between the internal capsule and the medial boundary of the LA. The regions and cells of interest for all recordings were located beneath the midpoint of the trunk spanning the LA horizontally.

### Whole-cell patch-clamp recordings

Whole-cell recordings were made using an Axopatch 200A amplifier or Multiclamp 700A (Molecular Devices, Sunnyvale, CA, USA). For the whole-cell voltage-clamp recordings, the recordings were obtained using a Cs-based internal solution containing (in mM) 100 Cs-gluconate, 0.6 EGTA, 10 HEPES, 5 NaCl, 20 TEA, 4 Mg-ATP, 0.3 Na-GTP and 3 QX314, with the pH adjusted to 7.2 with CsOH and the osmolarity adjusted to approximately 297 mmol/kg with sucrose. The cells used were classified as principal neurons based on the pyramidal shape of their somata. We included picrotoxin (100 μM) in our recording solution to isolate excitatory synaptic transmission and block feed-forward GABAergic inputs to the principal neurons in the LA. The pipette resistances ranged from 3.5 to 4.5 Mohm. IR-DIC-enhanced visual guidance was used to select neurons that were 3–4 cell layers below the surface of the 300-μm-thick slices, which were held at 32 ± 1°C. The neurons were voltage-clamped at –70 mV except during paired pulse low-frequency stimulation (ppLFS-pairing), and the various solutions were delivered to the slices via gravity-driven superfusion at a flow rate of 1.4~1.5 ml/min. The pipette series resistance was monitored throughout each experiment, and the data were discarded if it changed by >20%. Whole-cell currents were filtered at 1 kHz, digitized at up to 20 kHz, and stored on a microcomputer (Clampex 9 software, Molecular Devices, Sunnyvale, CA, USA). EPSCs were monitored following stimulation at 0.067 Hz. One or two neurons were recorded per animal (a single neuron per slice). In the ppLFS-pairing protocol, stimulation for 3 min at 3 Hz was performed using paired pulses (50 ms interpulse interval) while the neuron was clamped at –50 mV, as described previously (Clem and Huganir, 2013). Blockade of CP-AMPARs and mGluR1 was performed using NASPM (50 μM; Sigma-Aldrich) and LY367385 [(S)- (+)-α-amino-4-carboxy-2-methylbenzeneacetic acid] (100 μM; Tocris Bioscience). All EPSC amplitudes were normalized to an average of the baseline responses for the first 10 min and were expressed as percentages of the average baseline response. The percent inhibition by drugs (or vehicle) indicated difference in the average percentage of the responses between before and after drug (or vehicle) treatment. Therefore, the percent inhibition by NASPM in this study could be used to compare the amount of synaptic CP-AMPARs between before and after induction of LTD or depotentiation. The periods used to calculate these average responses were the 5 min immediately preceding drug treatment and the final 5 min after drug treatment, respectively. To prevent bias, we performed experiments in a blinded fashion. For improved visualization, the running averages of four data points were applied to the time-lapse experiments.

### Statistical analysis

**Figure 1:**
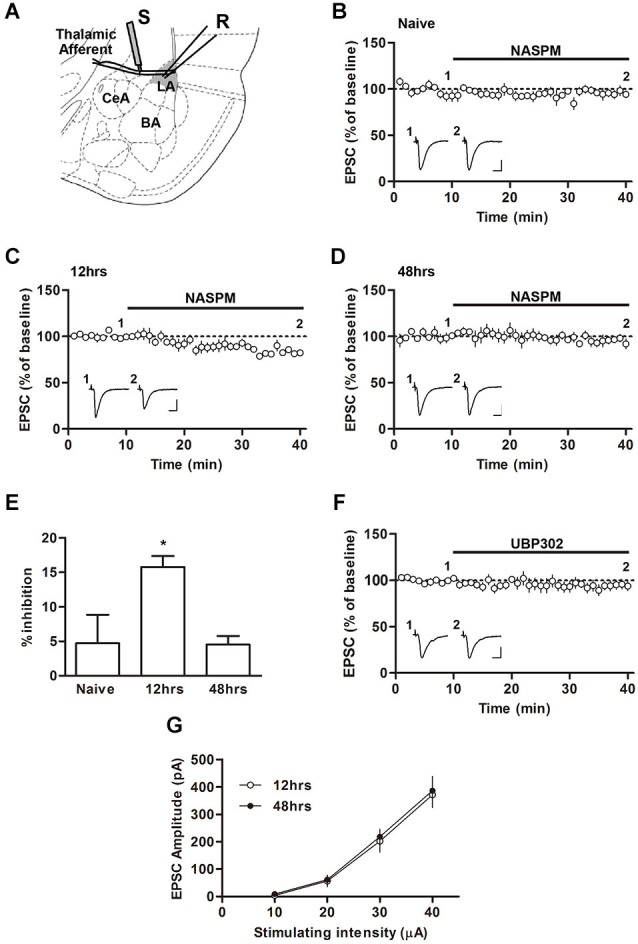
**Changes in the sensitivity to NASPM after fear conditioning. (A)** Diagram showing the positions of the stimulating and recording electrode. BA, basal nucleus of amygdala; CeA, central nucleus of amygdala; LA, lateral nucleus of amygdale; R, recording electrode; S, stimulating electrode. **(B–E)** NASPM (50 μM) treatment reduced the EPSC amplitude in the presence of D-AP5 (50 μM) in slices prepared 12 h after conditioning but not 48 h after conditioning or from naïve rats. **(F)** UBP302 treatment displayed no significant effects on the EPSC amplitude in slices prepared 12 h after conditioning. **(G)** The input-output relationship was not different between the groups of slices prepared 12 and 48 h after conditioning. * *p* < 0.05. Scale bars: 50 pA and 10 ms.

Between-group comparisons of the data were performed using either an unpaired *t*-test or one-way ANOVA with subsequent Newman-Keuls *post-hoc* comparison. A paired *t-test* was used to determine whether the post-treatment responses differed significantly from the baseline responses (Figures 1E, 3D). A *p*-value < 0.05 was considered to be statistically significant. The data from each neuron/slice were treated as independent samples. In all experiments using behaviorally trained rats, the data included samples from three or more animals.

**Figure 2:**
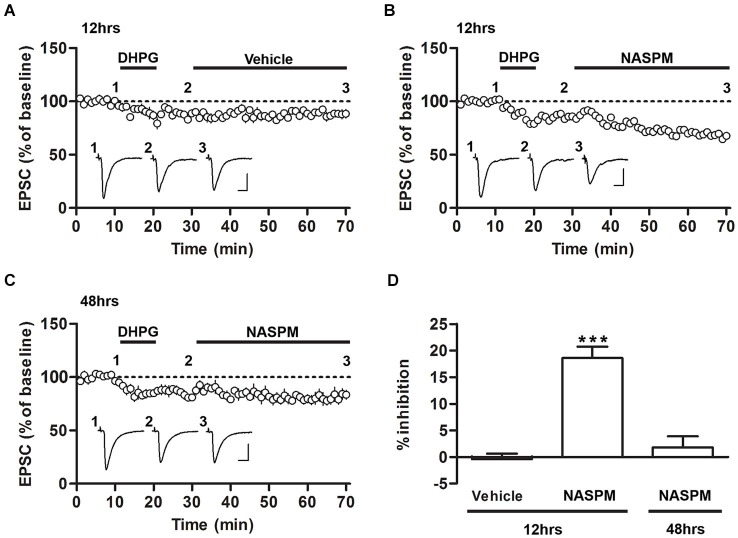
**Sensitivity to NASPM is maintained after DHPG-induced depotentiation. (A)** DHPG-induced depression was not significantly altered after vehicle treatment. The slices were prepared 12 h after conditioning. **(B)** NASPM treatment induced further depression after the onset of DHPG-induced depression. The slices were prepared 12 h after conditioning. **(C)** NASPM did not induce further changes in EPSCs after the onset of DHPG-induced depression. The slices were prepared 48 h after conditioning. All the experiments shown in this figure were performed in the presence of D-AP5 (50 μM). **(D)** A summary of the results shown in **(A–C)** (percent inhibition due to NASPM treatment for the three experiments). *** *p* < 0.001. Scale bars: 100 pA and 10 ms.

**Figure 3:**
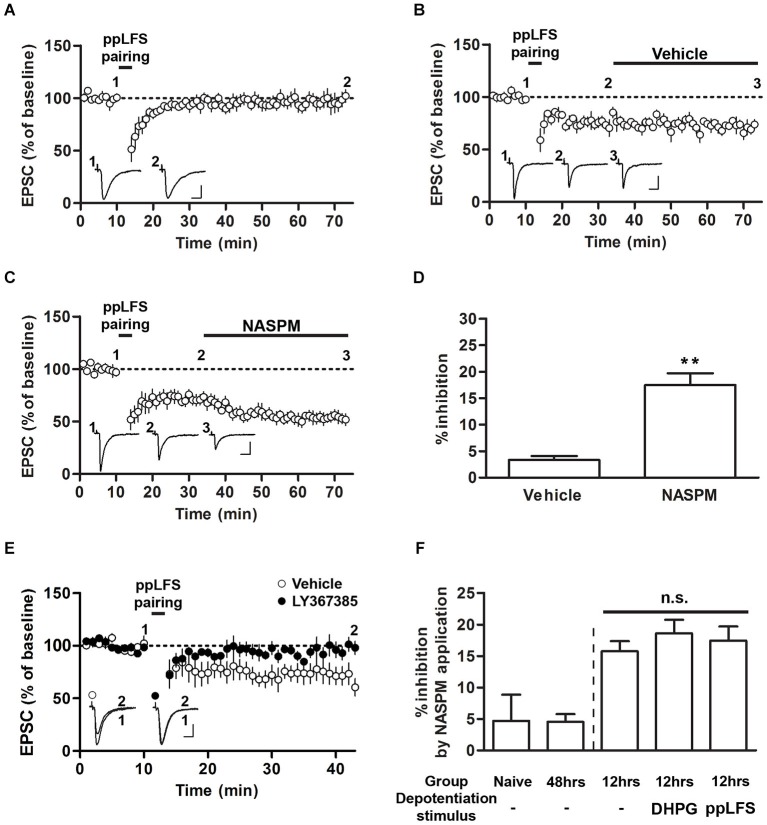
**Sensitivity to NASPM is maintained after ppLFS-pairing-induced depotentiation. (A)** ppLFS-pairing did not produce any significant changes in EPSCs. The slices were prepared from naïve rats. **(B)** pp-LFS-pairing produced synaptic depression, and vehicle treatment did not produce any further changes. The slices were prepared 12 h after conditioning. **(C)** ppLFS-pairing produced synaptic depression, and NASPM treatment produced further synaptic depression. The slices were prepared 12 h after conditioning. **(D)** Average percent inhibition due to NASPM (or vehicle) treatment for the experiments shown in B (vehicle) and C (NASPM). **(E)** ppLFS-pairing-induced depotentiation was blocked by the mGluR1 antagonist, LY367385. LY367385 (100 μM) or vehicle (aCSF) was present during the entire recording period. The slices were prepared 12 h after conditioning. **(F)** A summary of NASPM-induced inhibition in all the groups in which the effects of NASPM treatment were evaluated. ** *p* < 0.01. Scale bars: 50 pA and 10 ms.

## Results

### Synaptic expression of CP-AMPARs at T-LA synapses after fear conditioning

We first characterized the synaptic expression of CP-AMPARs according to the sensitivity of T-LA synaptic responses to NASPM, a CP-AMPAR antagonist, at various time points (12 and 48 h) after fear conditioning in rats. We measured AMPAR-mediated EPSCs at T-LA synapses in the presence of D-AP5 (50 μM) via whole-cell voltage-clamp recording in acute brain slices prepared from conditioned (or naïve) rats. A negligible level of CP-AMPARs was found in slices prepared from naïve rats, but a significant level of CP-AMPARs was detected 12 h after conditioning. The enhanced level of CP-AMPARs returned to baseline in slices prepared 48 h after conditioning (*F*_(2,13)_ = 4.787, *p* = 0.0319, one-way ANOVA; naïve, 4.70 ± 4.20%, *n* = 5; 12 h, 15.74 ± 1.6%, *n* = 4; 48 h, 4.52 ± 1.2%, *n* = 5; *p* < 0.05 for the 12 h group vs. the other groups, Newman-Keuls *post-hoc* test; Figures 1A–D), which was consistent with our previous study (Hong et al., 2013). Because NASPM has also been shown to inhibit kainate receptors (Koike et al., 1997; Cho et al., 2012), we examined whether UBP302 (10 μM), a specific antagonist of kainate receptors, blocked EPSCs at T-LA synapses when CP-AMPAR levels were elevated (12 h after conditioning). UBP302 displayed no significant effects on EPSCs at T-LA synapses in the presence of D-AP5 (50 μM) in slices prepared 12 h after fear conditioning (94.61 ± 4.10%, *n* = 5; *p* > 0.05, paired *t*-test; Figure 1E). In addition, we determined whether fear conditioning-induced synaptic potentiation was similar at those two time points (12 h vs. 48 h) after fear conditioning. There was no significant difference in the excitatory synaptic efficacy at T-LA synapses between the two groups (12 h after conditioning, 11.25 ± 1.57 pA/μA, *n* = 7; 48 h after conditioning, 11.84 ± 1.36 pA/μA, *n* = 9; *p* > 0.05, unpaired *t-test*; Figure 1F). Taken together, these results suggest that CP-AMPARs are transiently inserted into T-LA synapses after fear conditioning.

### CP-AMPARs are not removed from T-LA synapses via DHPG-induced depotentiation

We have previously shown that DHPG, an agonist of group I mGluRs, induces the depotentiation of fear conditioning-induced synaptic potentiation at T-LA synapses; that is, DHPG produces synaptic depression in slices prepared after fear conditioning but not in slices prepared from naïve rats or other controls (Kim et al., 2007; Hong et al., 2011). To examine whether CP-AMPARs are removed after induction of depotentiation, we monitored the changes in the sensitivity to NASPM after fear conditioning. Application of DHPG (100 μM, 10 min) successfully induced synaptic depression in the presence of D-AP5 (50 μM) in slices prepared 12 or 48 h after fear conditioning. After DHPG-induced depression had been stabilized, NASPM (or vehicle) was applied. NASPM treatment inhibited EPSCs significantly more than vehicle treatment when the CP-AMPAR levels were elevated (12 h after conditioning), but it did not exert a significant effect on EPSCs when CP-AMPARs were minimally expressed (48 h after conditioning) (*F*_(2,16)_ = 29.74, *p* < 0.001, one-way ANOVA; vehicle group-12 h after conditioning, −0.42 ± 1.04%, *n* = 5; NASPM group-12 h after conditioning, 18.59 ± 2.15%, *n* = 6; NASPM group-48 h after conditioning, 1.78 ± 2.11%, *n* = 6; *p* < 0.0001 for NASPM group-12 h after conditioning vs. the other groups, Newman-Keuls *post-hoc* test; Figures 2A–D). Thus, when CP-AMPARs are expressed at synapses, a significant proportion of CP-AMPARs appears to be retained even after the induction of depotentiation.

### CP-AMPARs are not removed from T-LA synapses via ppLFS-induced depotentiation

Previous studies have shown that ppLFS also induces depotentiation or LTD (Kim et al., 2007; Hong et al., 2009). We performed a protocol of ppLFS-pairing which was used by Clem and Huganir (2013). Unlike their findings that ppLFS-pairing induced LTD regardless of fear conditioning (either before or after conditioning), ppLFS-pairing produced no significant depression in slices from naïve rats (96.84 ± 7.33%, *n* = 5; *p* > 0.05, paired *t*-test; Figure 3A). Alternatively, ppLFS-pairing produced synaptic depression in slices prepared 12 h after conditioning, confirming the previously reported depotentiation of fear conditioning-induced synaptic potentiation (Figures 3B,C; Kim et al., 2007). After ppLFS-pairing-induced depression had been stabilized, NASPM (or vehicle) was applied. NASPM treatment inhibited EPSCs relative to vehicle treatment (vehicle, 3.33 ± 0.74%, *n* = 4; NASPM, 17.45 ± 2.24%, *n* = 7; *p* = 0.0013, unpaired *t-test*; Figures 3B–D). Therefore, similar to the results using DHPG, pre-existing CP-AMPARs appear to be largely retained after depotentiation. Because this particular result contradicts with the previous findings by Clem and Huganir (2010, 2013), it was necessary to confirm that ppLFS-induced depression in the present study shared the same induction requirement with that in the two previous studies (i.e., mGluR1-dependency). Indeed, application of the mGluR1 antagonist, LY367385, completely blocked ppLFS-pairing-induced depression (vehicle group, 69.89 ± 8.54%, *n* = 4; LY367385 group, 97.69 ± 5.89%, *n* = 7; *p* = 0.0223, unpaired *t-test*, Figure 3E).

Comparing the results from Figures 1–3 revealed that regardless of prior depotentiation, the primary factor that contributes to NASPM sensitivity at LA synapses is the duration after fear conditioning. NASPM-induced inhibition was minimal in naïve or 48 h, slices whereas in 12 h slices, NASPM-induced inhibition was prominent both before and after depotentiation, regardless of the depotentiation protocol (NASPM at baseline, NASPM after DHPG treatment and NASPM after pp-LFS) when the CP-AMPAR levels were elevated (12 h after conditioning) **(**NASPM at baseline (naïve rats), 4.70 ± 4.20%, *n* = 5; NASPM at baseline (48 h after conditioning), 4.52 ± 1.24%, *n* = 5; NASPM at baseline (12 h after conditioning), 15.74 ± 1.61%, *n* = 4; NASPM after DHPG treatment (12 h after conditioning), 18.59 ± 2.15% *n* = 6; NASPM after ppLFS (12 h after conditioning), 17.45 ± 2.24%, *n* = 7; *F*_(4,26)_ = 7.699, *p* = 0.0005, one-way ANOVA; *p* > 0.05 between the three groups prepared 12 h after conditioning, *p* < 0.05 for these three groups vs. the other two groups, Newman-Keuls *post-hoc* test; Figure 3F). Thus, our data suggest that fear conditioning produces transient insertion of CP-AMPARs into T-LA synapses, but these CP-AMPARs do not appear to be removed by depotentiation. The observation that depotentiation can be induced regardless of the expression of CP-AMPARs also suggests that depotentiation does not require the pre-existence of CP-AMPARs at synapses.

## Discussion

Our findings suggest that removal of CP-AMPARs from synapses does not contribute to the depotentiation of fear conditioning-induced synaptic potentiation. Under our experimental conditions, the sensitivity to NASPM is negligible before conditioning, increases 12 h after conditioning and returns to baseline 48 h after conditioning, suggesting that the synaptic expression of CP-AMPARs is minimal before conditioning and increases transiently after conditioning. We have also determined whether NASPM selectively inhibits CP-AMPARs, as it has also been shown to inhibit calcium-permeable kainate receptors. UBP302, a specific blocker of kainate receptors, does not inhibit T-LA EPSCs when CP-AMPARs are elevated (but see Cho et al., 2012), ruling out the possibility that the sensitivity to NASPM is due to inhibition of kainate receptor-mediated currents. Importantly, the sensitivity to NASPM does not change after induction of depotentiation, even when the synaptic expression of CP-AMPARs is elevated. This particular observation provides strong evidence that CP-AMPARs are not removed from T-LA synapses during depotentiation of fear conditioning-induced synaptic potentiation.

Our results provide an example in which group 1 mGluR-mediated synaptic depression does not require or promote CP-AMPAR removal. Instead, in T-LA synapses, it appears that mGluR1-dependent depotentiation leaves the pre-existing population of CP-AMPARs largely intact. This result is in contrast with previous reports in which the pre-existing CP-AMPAR content correlated to the extent of mGluR-LTD (Bellone and Lüscher, 2006), but our result is consistent with another report in which DHPG treatment induced synaptic depression regardless of prior CP-AMPAR expression (McCutcheon et al., 2011). Together, these results shed light on the downstream molecular mechanisms of mGluR-mediated synaptic depression.

Two recent studies have reported the contribution of CP-AMPARs to LTD at T-LA synapses (Clem and Huganir, 2010, 2013). In these studies, the magnitude of LTD has been shown to increase after fear conditioning, and the increased portion of LTD might represent depotentiation of fear conditioning-induced synaptic potentiation, although other interpretations are also possible (e.g., metaplastic enhancements in the magnitude of LTD after conditioning). In addition, the basal expression of synaptic CP-AMPARs is evident before fear conditioning, unlike our experimental conditions, which may involve a novel type of synaptic plasticity that is induced by calcium influx via pre-existing CP-AMPARs. Therefore, although the most parsimonious explanation for the increased magnitude of LTD after fear conditioning is the reversal of fear conditioning-induced synaptic potentiation, it remains possible that completely different types of synaptic plasticity (i.e., CP-AMPAR-dependent LTD whose magnitude can be enhanced after fear conditioning) are involved in these two studies. Therefore, distinct mechanisms may underlie LTD and depotentiation of fear conditioning-induced synaptic potentiation at T-LA synapses; indeed, these two types of plasticity (depotentiation and LTD) are known to underlie fear extinction and reconsolidation update (a variant of extinction that produces fear memory erasure), respectively (Kim et al., 2007; Clem and Huganir, 2010).

It is generally considered that in naïve animals (rats or mice), the synaptic expression of CP-AMPARs is negligible in the LA (Mahanty and Sah, 1998; Polepalli et al., 2010). Thus, it may appear odd to detect a relatively large level of CP-AMPARs under baseline conditions in the two previous studies (Clem and Huganir, 2010, 2013). It will be interesting to determine which conditions affect the amount of synaptic CP-AMPARs in the experimental subjects (see also Whitehead et al., 2013).

Our previous study (Kim et al., 2007) has shown that ppLFS-induced depotentiation (or LTD) is dependent on both mGluR and NMDAR activity at T-LA synapses. Similarly, Clem and Huganir have also reported that ppLFS-induced LTD is dependent on both mGluR and NMDAR activity at T-LA synapses (Clem and Huganir, 2010, 2013). In these three studies, ppLFS-induced LTD (or depotentiation) was completely blocked either by mGluR antagonists or by NMDAR antagonists unlike the case of the hippocampal LTD in which each antagonist partially blocked LTD (Oliet et al., 1997). Furthermore, Clem and Huganir (2013) has shown that LFS-induced LTD is dependent on NMDARs, but not on mGluRs at T-LA synapses, and that LFS-induced LTD is produced via mechanisms that were completely different from those underlying ppLFS-induced LTD. Therefore, it is possible that ppLFS induces a unique form of LTD, which depends on both mGluRs and NMDARs, at T-LA synapses, and that these two types of receptors merge on the same intracellular signaling pathway.

There have been previous studies showing the existence of several forms of LTD in the lateral amygdala. Low-frequency or theta-frequency stimulation has been shown to produce LTD at naive synapses in the lateral amygdala (Heinbockel and Pape, 2000; Dalton et al., 2012; Clem and Huganir, 2013). It remains to be elucidated whether these forms of LTD share similar mechanisms or not. Moreover, other new forms of LTD may coexist at lateral amygdala synapses and each of them may play a unique role in a distinct physiological process.

In summary, the present study has provided strong evidence that the removal of CP-AMPARs from synapses does not contribute to the depotentiation of fear conditioning-induced synaptic potentiation. This conclusion is consistent with our and other previous studies showing that extinction (whose cellular substrate is the depotentiation of fear conditioning-induced synaptic potentiation) does not involve CP-AMPAR removal (Clem and Huganir, 2013; Lee et al., 2013). In contrast, removal of CP-AMPARs via LTD at T-LA synapses has been proposed as a mechanism underlying reconsolidation update. Thus, the molecular mechanisms underlying LTD may differ from those underlying depotentiation of fear conditioning-induced synaptic potentiation at T-LA synapses.

## Conflict of interest statement

The authors declare that the research was conducted in the absence of any commercial or financial relationships that could be construed as a potential conflict of interest.

## References

[B1] Ahmadian G. Ju W. Liu L. Wyszynski M. Lee S. H. Dunah A. W. (2004). Tyrosine phosphorylation of GluR2 is required for insulin-stimulated AMPA receptor endocytosis and LTD. EMBO J. 23, 1040–1050 10.1038/sj.emboj.7600126 14976558 PMC380981

[B2] Bellone C. Lüscher C. (2005). mGluRs induce a long-term depression in the ventral tegmental area that involves a switch of the subunit composition of AMPA receptors. Eur. J. Neurosci. 21, 1280–1288 10.1111/j.1460-9568.2005.03979.x 15813937

[B3] Bellone C. Lüscher C. (2006). Cocaine triggered AMPA receptor redistribution is reversed in vivo by mGluR-dependent long-term depression. Nat. Neurosci. 9, 636–641 10.1038/nn1682 16582902

[B4] Cho J. H. Bayazitov I. T. Meloni E. G. Myers K. M. Carlezon W. A. Jr. Zakharenko S. S. (2012). Coactivation of thalamic and cortical pathways induces input timing-dependent plasticity in amygdala. Nat. Neurosci. 15, 113–122 10.1038/nn.2993 22158512 PMC3245819

[B5] Chung H. J. Steinberg J. P. Huganir R. L. Linden D. J. (2003). Requirement of AMPA receptor GluR2 phosphorylation for cerebellar long-term depression. Science 300, 1751–1755 10.1126/science.1082915 12805550

[B6] Clem R. L. Huganir R. L. (2010). Calcium-permeable AMPA receptor dynamics mediate fear memory erasure. Science 330, 1108–1112 10.1126/science.1195298 21030604 PMC3001394

[B7] Clem R. L. Huganir R. L. (2013). Norepinephrine enhances a discrete form of long-term depression during fear memory storage. J. Neurosci. 33, 11825–11832 10.1523/JNEUROSCI.3317-12.2013 23864672 PMC3713724

[B8] Collingridge G. L. Peineau S. Howland J. G. Wang Y. T. (2010). Long-term depression in the CNS. Nat. Rev. Neurosci. 11, 459–473 10.1038/nrn2867 20559335

[B9] Dalton G. L. Wu D. C. Wang Y. T. Floresco S. B. Phillips A. G. (2012). NMDA GluN2A and GluN2B receptor splay separate roles in the induction of LTP and LTD in the amygdala and in the acquisition and extinction of conditioned fear. Neuropharmacology 62, 797–806 10.1016/j.neuropharm.2011.09.001 21925518

[B10] Heinbockel T. Pape H. C. (2000). Input-specific long-term depression in the lateral amygdala evoked by theta frequency stimulation. J. Neurosci. 20, RC68 10729357 10.1523/JNEUROSCI.20-07-j0002.2000 PMC6772226

[B11] Hong I. Kim J. Kim J. Lee S. Ko H. G. Nader K. (2013). AMPA receptor exchange underlies transient memory destabilization on retrieval. Proc. Natl. Acad. Sci. U S A 110, 8218–8223 10.1073/pnas.1305235110 23630279 PMC3657785

[B12] Hong I. Kim J. Lee J. Park S. Song B. Kim J. (2011). Reversible plasticity of fear memory-encoding amygdala synaptic circuits even after fear memory consolidation. PLoS One 6:e24260 10.1371/journal.pone.0024260 21949700 PMC3176280

[B13] Hong I. Song B. Lee S. Kim J. Kim J. Choi S. (2009). Extinction of cued fear memory involves a distinct form of depotentiation at cortical input synapses onto the lateral amygdala. Eur. J. Neurosci. 30, 2089–2099 10.1111/j.1460-9568.2009.07004.x 20128847

[B14] Kelly L. Farrant M. Cull-Candy S. G. (2009). Synaptic mGluR activation drives plasticity of calcium-permeable AMPA receptors. Nat. Neurosci. 12, 593–601 10.1038/nn.2309 19377472

[B15] Kim J. Lee S. Park K. Hong I. Song B. Son G. H. (2007). Amygdala depotentiation and fear extinction. Proc. Natl. Acad. Sci. U S A 104, 20955–20960 10.1073/pnas.0710548105 18165656 PMC2409248

[B16] Klausnitzer J. Kulla A. Manahan-Vaughan D. (2004). Role of the group III metabotropic glutamate receptor in LTP, depotentiation and LTD in the dentate gyrus of freely moving rats. Neuropharmacology 46, 160–170 10.1016/j.neuropharm.2003.09.019 15080077

[B17] Knapska E. Maren S. (2009). Reciprocal patterns of c-fos expression in the medial prefrontal cortex and amygdala after extinction and renewal of conditioned fear. Learn. Mem. 16, 486–493 10.1101/lm.14 19633138 PMC2726014

[B18] Koike M. Iino M. Ozawa S. (1997). Blocking effect of 1-naphthyl acetyl spermine on Ca(2+)-permeable AMPA receptors in cultured rat hippocampal neurons. Neurosci. Res. 29, 27–36 10.1016/s0168-0102(97)-9 9293490

[B19] Kulla A. Reymann K. G. Manahan-Vaughan D. (1999). Time-dependent induction of depotentiation in the dentate gyrus of freely moving rats: involvement of group 2 metabotropic glutamate receptors. Eur. J. Neurosci. 11, 3864–3872 10.1046/j.1460-9568.1999.00807.x 10583475

[B20] LeDoux J. E. (2000). Emotion circuits in the brain. Annu. Rev. Neurosci. 23, 155–184 10.1146/annurev.neuro.23.1.155 10845062

[B22] Lee S. Song B. Kim J. Park J. Hong I. An B. (2013). GluA1 phosphorylation at serine 831 in the lateral amygdala is required for fear renewal. Nat. Neurosci. 16, 1436–1444 10.1038/nn.3491 23974710

[B21] Lin C.-H. Lee C.-C. Gean P.-W. (2003). Involvement of a calcineurin cascade in amygdala depotentiation and quenching of fear memory. Mol. Pharmacol. 63, 44–52 10.1124/mol.63.1.44 12488535

[B23] Lin C.-H. Lee C.-C. Huang Y.-C. Wang S.-J. Gean P.-W. (2005). Activation of group II metabotropic glutamate receptors induces depotentiation in amygdala slices and reduces fear-potentiated startle in rats. Learn. Mem. 12, 130–137 10.1101/lm.85304 15774944 PMC1074330

[B24] Loweth J. A. Scheyer A. F. Milovanovic M. LaCrosse A. L. Flores-Barrera E. Werner C. T. (2013). Synaptic depression via mGluR1 positive allosteric modulation suppresses cue-induced cocaine craving. Nat. Neurosci. 17, 73–80 10.1038/nn.3590 24270186 PMC3971923

[B25] Lüscher C. Huber K. M. (2010). Group 1 mGluR-dependent synaptic long-term depression: mechanisms and implications for circuitry and disease. Neuron 65, 445–459 10.1016/j.neuron.2010.01.016 20188650 PMC2841961

[B26] Mahanty N. K. Sah P. (1998). Calcium-permeable AMPA receptors mediate long-term potentiation in interneurons in the amygdala. Nature 394, 683–687 10.1038/29312 9716132

[B27] Maren S. Quirk G. J. (2004). Neuronal signalling of fear memory. Nat. Rev. Neurosci. 5, 844–852 10.1038/nrn1535 15496862

[B28] McCutcheon J. E. Loweth J. A. Ford K. A. Marinelli M. Wolf M. E. Tseng K. Y. (2011). Group I mGluR activation reverses cocaine-induced accumulation of calcium-permeable AMPA receptors in nucleus accumbens synapses via a protein kinase C-dependent mechanism. J. Neurosci. 31, 14536–14541 10.1523/JNEUROSCI.3625-11.2011 21994370 PMC3220940

[B29] McKernan M. G. Shinnick-Gallagher P. (1997). Fear conditioning induces a lasting potentiation of synaptic currents in vitro. Nature 390, 607–611 10.1038/37605 9403689

[B30] Moult P. R. Gladding C. M. Sanderson T. M. Fitzjohn S. M. Bashir Z. I. Molnar E. (2006). Tyrosine phosphatases regulate AMPA receptor trafficking during metabotropic glutamate receptor-mediated long-term depression. J. Neurosci. 26, 2544–2554 10.1523/jneurosci.4322-05.2006 16510732 PMC6793648

[B31] Myers K. M. Davis M. (2007). Mechanisms of fear extinction. Mol. Psychiatry 12, 120–150 10.1038/sj.mp.4001939 17160066

[B32] Oliet S. H. Malenka R. C. Nicoll R. A. (1997). Two distinct forms of long-term depression coexist in CA1 hippocampal pyramidal cells. Neuron 18, 969–982 10.1016/s0896-6273(00)80336-0 9208864

[B33] Polepalli J. S. Sullivan R. K. Yanagawa Y. Sah P. (2010). A specific class of interneuron mediates inhibitory plasticity in the lateral amygdala. J. Neurosci. 30, 14619–14629 10.1523/JNEUROSCI.3252-10.2010 21048119 PMC6633620

[B34] Rogan M. T. Stäubli U. V. LeDoux J. E. (1997). Fear conditioning induces associative long-term potentiation in the amygdala. Nature 390, 604–607 10.1038/37601 9403688

[B35] Wagner J. J. Alger B. E. (1996). Homosynaptic LTD and depotentiation: do they differ in name only? Hippocampus 6, 24–29 10.1002/(sici)1098-1063(1996)6:1<24::aid-hipo5>3.0.co;2-7 8878738

[B36] Whitehead G. Jo J. Hogg E. L. Piers T. Kim D. H. Seaton G. (2013). Acute stress causes rapid synaptic insertion of Ca2+-permeable AMPA receptors to facilitate long-term potentiation in the hippocampus. Brain 136(Pt. 12), 3753–3765 10.1093/brain/awt293 24271563 PMC3859225

